# Food items contributing most to variation in antioxidant intake; a cross-sectional study among Norwegian women

**DOI:** 10.1186/1471-2458-14-45

**Published:** 2014-01-16

**Authors:** Samera Azeem Qureshi, Annette Christin Lund, Marit Bragelien Veierød, Monica Hauger Carlsen, Rune Blomhoff, Lene Frost Andersen, Giske Ursin

**Affiliations:** 1 Department of Nutrition, Institute of Basic Medical Sciences, University of Oslo, Oslo, Norway; 2 Department of Biostatistics, Institute of Basic Medical Sciences, University of Oslo, Oslo, Norway; 3 Division of Cancer, Surgery and Transplantation, Oslo University Hospital, Oslo, Norway; 4 Cancer Registry of Norway, P.O. Box 5313, Majorstuen 0304, Oslo, Norway; 5 Department of Preventive Medicine, University of Southern California, Los Angeles, CA, USA

**Keywords:** Antioxidants, Epidemiology, Nutrition, Fruits, Coffee, Vegetables

## Abstract

**Background:**

Fruit and vegetable intake has been found to reduce the risk of cardiovascular disease, certain types of cancer and diabetes mellitus. It is possible that antioxidants play a large part in this protective effect. However, which foods account for the variation in antioxidant intake in a population is not very clear. We used food frequency data from a population-based sample of women to identify the food items that contributed most to the variation in antioxidant intake in Norwegian diet.

**Methods:**

We used data from a study conducted among participants in the Norwegian Breast Cancer Screening Program (NBCSP), the national program which invites women aged 50–69 years to mammographic screening every 2 years. A subset of 6514 women who attended the screening in 2006/2007 completed a food frequency questionnaire (FFQ). Daily intake of energy, nutrients and antioxidant intake were estimated. We used multiple linear regression analysis to capture the variation in antioxidant intake.

**Results:**

The mean (SD) antioxidant intake was 23.0 (8.5) mmol/day. Coffee consumption explained 54% of the variation in antioxidant intake, while fruits and vegetables explained 22%. The twenty food items that contributed most to the total variation in antioxidant intake explained 98% of the variation in intake. These included different types of coffee, tea, red wine, blueberries, walnuts, oranges, cinnamon and broccoli.

**Conclusions:**

In this study we identified a list of food items which capture the variation in antioxidant intake among these women. The major contributors to dietary total antioxidant intake were coffee, tea, red wine, blueberries, walnuts, oranges, cinnamon and broccoli. These items should be assessed in as much detail as possible in studies that wish to capture the variation in antioxidant intake.

## Background

Fruit and vegetable intake are associated with a reduced risk of cardiovascular disease and possibly with reduced risk of several cancers [1-5]. The exact constituents responsible for the protective effect of fruits and vegetables are not known. However, several lines of evidence suggest that antioxidants, i.e. compounds that dampen oxidative stress or eliminate excess reactive oxygen and nitrogen species (ROS and RNS), may play a major role [2,3,6-9].

ROS/RNS are small reactive molecules, which are produced as a result of normal metabolism [10], but also occur as active compounds in immune responses or signalling pathways [11]. Increased production of ROS that cannot be handled by the cells is called oxidative stress [10]. This can damage the DNA and is therefore believed to play a role in both cardiovascular disease and several cancers [12]. Antioxidants can eliminate free radicals and other reactive oxygen and nitrogen species [8,13].

Total antioxidant capacity measures in a single value all antioxidants present in samples of food. Several assays have been used to assess the total antioxidant content of foods, e.g. the 6-hydroxy-2,5,7,8-tetramethylchroman-2-carboxylic acid (Trolox) equivalent antioxidant capacity (TEAC) assay [14], the ferric-reducing ability of plasma (FRAP) [15] and the oxygen radical absorbance capacity assay (ORAC) assay [16]. The present study used a database where antioxidant content of numerous foods had been measured using the FRAP method [17].

The antioxidant capacity measured by the FRAP method determines the total concentration of redox-active compounds above a certain cut off reduction potential. In addition to the potential role as direct antioxidants, many of these antioxidants are also potent inducers of gene transcriptions related to the endogenous antioxidant defence. In fact, recent studies suggest that this indirect effect of plant antioxidants may be more relevant than a direct effect on ROS and RNS [18]. Blood cell gene expression associated with cellular stress defence is modulated by antioxidant-rich food in a randomised controlled clinical trial of male smokers [18].

Foods high in antioxidants include fruits, berries, vegetables, spices, coffee and tea. From a clinical nutrition point of view when assessing an individual patient’s diet, or when giving advice to an individual on how to increase antioxidant intake, it would be important to focus on good antioxidant sources in order to increase the person’s total intake of antioxidants. Thus a clinical nutritionist would primarily be interested in identifying individual food items rich in antioxidants, and on foods that are commonly eaten. One example would be oranges, which are high in antioxidants and commonly eaten. The nutritionist would probably give the same advice to every patient. However, to study the epidemiology of various diseases, it is more important to identify the dietary items that explain the *variation* in intake of antioxidants between individuals in a population. If those food items are included in dietary assessments, researchers can better capture the between person variation, and thus assess relative risk of disease in individuals high in antioxidant intake compared to those low in antioxidant intake [19]. There will often be overlap in which items explain the total intake, and which explain the variation in intake between individuals. However, there can also be important differences. If every individual in a population eats an orange a day, then oranges can contribute substantially to the *total* intake in nutrients, but will not explain the *variation* in intake between individuals, since everyone eats one a day.

A previous study estimated the contribution of intake of coffee, tea, wine, cereal, fruit and vegetables to the *total* antioxidant intake in a sample of 2672 Norwegian men and women [20]. However, this study did not determine the food items that contributed to the *variation* in antioxidant intake between individuals, and the questionnaire used did not include questions about some food items high in antioxidants, assumed to be prevalent in a Northern European diet. We decided to expand on these findings and conducted a study using a more comprehensive food frequency questionnaire (FFQ) with detailed questions on items high in antioxidants, including chocolate, nuts and seeds, berries, herbs and spices. We aimed at identifying the individual food items contributing most to the variation in antioxidant (in terms of FRAP content) intake in the Norwegian diet.

## Methods

### Study sample

We used data from the Norwegian Breast Cancer Screening Program (NBCSP), a governmentally funded national screening program [21]. Women aged 50–69 years are invited to a bilateral 2-view mammogram biennially. The participation rate in the mammographic screening program is about 77% [22], with about 250,000 women invited per year. In 2006/2007, a standard risk factor questionnaire was enclosed with the invitation letter for screening. Around 11,592 women agreed to participate in 2006 and 55,935 in 2007. A consent form and a FFQ were sent to a random sample of 10,000 of these women in 2008. Out of these 6928 women returned the completed dietary questionnaire within the aliquoted time frame. We excluded 213 women with very high (>15,000 kilojoules (kJ)) and 17 women with very low (< 2100 kJ) energy intake per day. We also excluded women aged less than 50 years (*n* = 72) or more than 69 years (*n* = 2), women who reported they weighed less than 30 kg (*n* = 77) or more than 170 kg (*n* = 5), and women who reported height less than 125 cm (*n* = 28). A total of *n* = 6514 women were included in this analysis. All participants signed an informed consent. The project was approved by the regional ethics committee and the Norwegian Data Inspectorate.

### FFQ

The questionnaire was based on a validated, 16 paged semi-quantitative FFQ, which included questions on 270 dietary items [17,23,24]. The original questionnaire was designed to cover 100% of the total energy intake of the population [17,24]. For our study the questionnaire was expanded in order to capture foods with high antioxidant content, based on an extensive screening of antioxidant content in foods and beverages consumed in Norway [25]. Additional questions were added concerning intake of several food categories. In detail, 19 questions about berries, 4 questions about fruit, 6 questions about vegetables, 2 questions about chocolate, 3 questions about coffee and 2 questions about tea were added. Furthermore, 10 questions were added about nuts and seeds and 27 questions about spices and herbs [25]. The respondents had to choose from options for frequency of consumption of particular food items ranging from several times a day to never/seldom. The portion sizes were based on: slices, glasses, cups, pieces, spoons and teaspoons. When a respondent only answered about the frequency but not portion size, the food item was given the smallest portion size. If only the amount of the food item was given, the frequency was set to zero. The dietary questions covered 12 pages of the questionnaire whereas the last 4 pages included questions on dietary supplements, smoking, physical activity, illnesses and medication. More details on the questionnaire have been reported earlier [25]. For some seasonal food items, such as berries, there were two questions to capture the intake; one regarding intake during the time period when the berries are in season, and the other regarding the intake during the other 10 months of the year. We combined the two variables to form one single variable, in order to estimate the average intake during the whole year.

### Dietary assessment

Daily intake of energy, nutrients and antioxidants were computed using software developed at the Department of Nutrition, University of Oslo (KBS version 4.9, 2008). We used the food database KBS AE- 07, which was based on the 2006 edition of the Norwegian food composition table (http://www.matvaretabellen.no). The database was supplemented with antioxidant content in foods that were measured using FRAP method expressed as mmol/day [17,26].

### Statistical analysis

Total antioxidant intake (FRAP values expressed as mmol/day) and all the food items in g/day (fruits, vegetables, spices, herbs, drinks) were analysed as continuous variables. We used forward stepwise multiple regression analysis to select the variables that best explained the variation in antioxidant intake [27]. We report both the unstandardized and the standardized regression coefficients. The standardized regression coefficients describe the change in the dependent variable in units of standard deviations for a one standard deviation change in the independent variable, and enables comparison of the regression coefficients. To identify the top food items explaining variation in intake, we present the list of the food items ranked according to the beta co-efficient in descending order.

In our analyses we controlled for the following covariates, all modelled as continuous variables; age at the time of mammographic screening, body mass index (BMI) calculated by dividing self-reported weight (kg) by height squared (m^2^), and energy intake in kJ. The variables were forced into the stepwise regressions.

As the residuals from the regression model did not satisfy the normality assumption, we log-transformed all variables. This resulted in the normality assumptions being met, but the results were not different from the non-transformed model, specifically, the order of the food items was the same in the two models. For simplicity in interpreting the coefficients, we therefore present the results from the non-transformed model.

We ran a forward stepwise regression and used a *P* for entry into the regression model of < 0.05 and a *P* for removal of > 0.10. This was done as follows: We first ran separate models within each food group (fruits, vegetables, berries, herbs, spices, nuts and drinks) with adjustments for the covariates listed above to identify which individual food items were the most important predictors within a food group. In the final model we included all the food items from each food group analysis that satisfied the above criteria, while adjusting for the same covariates. We also did a number of sensitivity analyses where we reran the analyses using several higher values for *P* for entry and *P* for removal. In these analyses we used *P* for entry of < 0.10 and < 0.15, and *P* for removal of > 0.15 and > 0.20. However, this did not alter the sequence of the food items according to the antioxidant content, so these results are not presented. All analyses were performed using the software package STATA v. 11 (StataCorp, College Station, TX).

## Results

The characteristics of the study sample are described in Table 1. The mean (standard deviation) age of the 6514 participants was 57.8 (4.5) years, and their mean BMI was 29.8 (5.1) kg/m^2^. The mean energy intake was 8615 (2216) kJ/day and the mean antioxidant intake was 23.0 (8.5) mmol/day.

**Table 1:** Characteristics of the study participants (n = 6514)

**Variables**	**Mean (SD)**	**Range (min–max)**	**Median**	Interquartile range (P25–P75)*
Age (years)	57.8 (4.5)	50–69	58	54–62
Energy (kJ/day)	8615 (2216)	2166–14993	8445.5	7049–10007
Weight (kg)	71.0 (12.4)	38–170	59	63–78
Height (cm)	166.4 (5.6)	140–190	167	163–170
BMI (kg/m^2^)	29.8 (5.1)	17.0–60.0	29.5	27–33
Antioxidant intake (mmol/day)	23.0 (8.5)	0.9–88.0	22.3	17–28
Fruits (g/day)	295 (175)	0–1742	272	175–387
Berries (g/day)	12 (15.5)	0–254	7.2	3–15
Vegetables (g/day)	288 (152)	0–1847	262	188–355

*P25, 25th percentile; P75, 75th percentile.

Fruits and vegetables contributed 22%, berries contributed 18% and coffee consumption contributed 54% to the variation in antioxidant intake between individuals in our study. The fruits without berries explained around 17% of the variation in antioxidant intake, whereas the vegetables also explained roughly 17% of the variation. When both fruits and vegetables were included in the model they explain 22% of the variation in antioxidant intake.

Table 2 shows the results for the top fruits, berries and vegetables contributing to the variation in antioxidant intake, while Tables 3, 4 and 5 show the results for herbs, nuts and drinks, respectively. The results are sorted in descending order according to the standardized regression coefficients. Among fruits the most important contributors to variation in antioxidant intake were oranges and apples (Table 2), blueberries explained the highest variation in antioxidant intake among berries, whereas tomatoes, bell pepper and salad were at the top of the list among vegetables. Among herbs and spices garlic and cinnamon explained the highest variation in antioxidant intake (Table 3), and among nuts walnuts were at the top of the list (Table 4). Among the list of drinks filtered coffee, green tea and boiled coffee were at the top (Table 5).

**Table 2:** Regression coefficients* of top fruits, berries and vegetables contributing to antioxidant intake (*n* **= 6514)**

	**Unstandardized coefficients**		**Standardized coefficients**	
**Fruits (g/day)**	**Estimate**	**Std. error**	**Estimate**	***P *** **value**
Oranges	0.01	< 0.01	0.11	< 0.01
Apples	0.01	< 0.01	0.05	< 0.01
Apricot	0.04	< 0.01	0.05	< 0.01
Clementine	0.01	< 0.01	0.04	< 0.01
Kiwi	0.02	< 0.01	0.04	< 0.01
Peach	0.01	< 0.01	0.03	0.01
Pomegranate	0.07	0.02	0.03	0.01
Prune	0.02	0.01	0.03	0.01
**Berries (g/day)**				
Blueberries	0.10	0.01	0.11	< 0.01
Cherries	0.12	0.02	0.07	< 0.01
Cranberries	0.16	0.04	0.05	< 0.01
Blackberries	0.18	0.04	0.05	< 0.01
Cloudberries	0.29	0.12	0.03	0.01
Rosehips	0.32	0.14	0.02	0.03
**Vegetables (g/day)**				
Tomatoes	0.02	< 0.01	0.06	0.01
Bell pepper	0.05	0.01	0.06	0.01
Salad	0.01	< 0.01	0.06	0.01
Broccoli	0.01	< 0.01	0.05	0.01
Onions	0.04	0.01	0.03	0.01
Turnips	0.02	0.01	0.03	0.02
Cauliflower	0.01	0.01	0.03	0.04
Cabbage	0.05	0.02	0.03	0.03
Corn	−0.03	0.02	−0.02	0.04

Sorted in descending order according to standardized coefficients.

*adjusted for age, BMI and energy intake.

**Table 3:** Regression coefficients* of top six herbs contributing to antioxidant intake (*n* **= 6514)**

	**Unstandardized coefficients**		**Standardized coefficients**	
**Herbs (g/day)**	**Estimate**	**Std. error**	**Estimate**	***P *** **value**
Garlic	0.21	0.03	0.08	0.01
Cinnamon	1.24	0.19	0.08	0.01
Black pepper	0.57	0.15	0.05	0.01
Dried rosemary	3.43	1.17	0.03	0.01
Fresh dill	4.83	2.00	0.03	0.02
Fresh peppermint	−6.77	2.80	−0.03	0.02

Sorted in descending order according to standardized coefficients.

*adjusted for age, BMI and energy intake.

**Table 4:** Regression coefficients* of top five nuts contributing to antioxidant intake (*n* **= 6514)**

	**Unstandardized coefficients**		**Standardized coefficients**	
**Nuts (teaspoons/day)**	**Estimate**	**Std. error**	**Estimate**	***P *** **value**
Walnuts	2.20	0.21	0.12	< 0.01
Sunflower seeds	0.50	0.18	0.03	0.01
Cashew nuts	0.44	0.22	0.03	0.04
Pine seeds	1.34	0.65	0.02	0.04
Almonds	0.26	0.15	0.02	0.08

Sorted in descending order according to standardized coefficients.

*adjusted for age, BMI and energy intake.

**Table 5:** Regression coefficients* of top 15 drinks contributing to antioxidant intake (*n* **= 6514)**

	**Unstandardized coefficients**		**Standardized coefficients**	
**Drinks (g/day)**	**Estimate**	**Std. error**	**Estimate**	***P *** **value**
Coffee, filtered	0.03	< 0.01	0.80	< 0.01
Green tea	0.02	< 0.01	0.41	< 0.01
Coffee, boiled	0.02	< 0.01	0.40	< 0.01
Regular tea	0.01	< 0.01	0.33	< 0.01
Coffee, instant	0.02	< 0.01	0.30	< 0.01
Red wine	0.02	< 0.01	0.20	< 0.01
Coffee, cafe latte	0.02	< 0.01	0.15	< 0.01
Mixed rosehip/orange juice	0.02	< 0.01	0.14	< 0.01
Herbal tea	0.01	< 0.01	0.11	< 0.01
Coffee, espresso	0.14	< 0.01	0.11	< 0.01
Blueberry juice	0.02	< 0.01	0.10	< 0.01
Coffee, cappuccino	0.02	< 0.01	0.08	< 0.01
Orange juice	0.01	< 0.01	0.07	< 0.01
Cranberry juice	0.01	< 0.01	0.02	< 0.01

Sorted in descending order according to standardized coefficients.

*adjusted for age, BMI and energy intake.

Table 6 reports the regression coefficients from the final model where all the food items were included with antioxidant intake as the dependent variable. The top 20 food items in explaining the variation in antioxidant intake included different types of coffee and tea, as well as blueberries, walnuts, cinnamon and broccoli. These 20 items explained 98% of the total variation in antioxidant intake. When we reran the model without including coffee and tea, the rest of the food items (fruits, vegetables, berries, spices and nuts) explained only 22% of the variation in antioxidant intake.

**Table 6:** Regression coefficients* of top 20 food items contributing to antioxidant intake (*n* **= 6514)**

	**Unstandardized coefficients**		**Standardized coefficients**	
**Food items (g/day)**	**Estimate**	**Std. error**	**Estimate**	***P *** **value**
Coffee, filtered	0.03	< 0.01	0.81	< 0.01
Coffee, boiled	0.02	< 0.01	0.40	< 0.01
Green tea	0.01	< 0.01	0.40	< 0.01
Regular tea	0.01	< 0.01	0.33	< 0.01
Coffee, instant	0.02	< 0.01	0.28	< 0.01
Red wine	0.02	< 0.01	0.19	< 0.01
Coffee, cafe latte	0.02	< 0.01	0.15	< 0.01
mixed rosehip/orange juice	0.02	< 0.01	0.14	< 0.01
Coffee, espresso	0.14	< 0.01	0.11	< 0.01
Blueberry juice	0.02	< 0.01	0.10	< 0.01
Blueberries	0.10	< 0.01	0.10	< 0.01
Walnuts	1.60	0.02	0.10	< 0.01
Coffee, cappuccino	0.02	< 0.01	0.08	< 0.01
Herbal tea	< 0.01	< 0.01	0.08	< 0.01
Orange juice	0.01	< 0.01	0.08	< 0.01
Orange	0.01	< 0.01	0.07	< 0.01
Cinnamon	0.83	0.02	0.05	< 0.01
Broccoli	0.01	< 0.01	0.05	< 0.01
Cherries	0.02	< 0.01	0.04	< 0.01
Clementine	0.01	< 0.01	0.03	< 0.01

Sorted in descending order according to standardized beta coefficients.

*adjusted for age, BMI and energy intake.

We also analysed separately non-coffee drinkers and non-tea drinkers, and also those who drank neither of the two. Among the non-coffee drinkers (*n* = 648) the top 20 food items contributed 98% of the variation in antioxidant intake, and green tea was at the top of the list followed by regular tea, red wine and blueberry juice. In case of non-tea drinkers (*n* = 2620) the top 20 food items contributed 99% of the variation in antioxidant intake, and filtered coffee was at the top of the list followed by boiled, instant coffee and red wine. In the third group (non-coffee and non-tea drinkers, *n* = 141) the top 20 food items contributed 95% of the variation in antioxidant intake, and blueberries were at the top followed by blueberry juice, red wine and mixed rosehip/orange juice (results not shown).

We also did a sensitivity analysis where we analysed data in young (aged 50–59) as well as in older (age 60–69) women. There was not much difference in the sequence of the top food items that contributed most to the variation in antioxidant intake (coffee, tea, red wine, blueberries, oranges, broccoli, and cinnamon). Cherries were at number 16 in the list among younger women, whereas among the older women they were at number 20. In both groups the top twenty food items explained 98% of the variation.

Similarly, we reran analyses among the women with BMI ≤ 25.0 and the overweight women (BMI > 25.0). In both groups filtered coffee was at the top of the list of the 20 food items. However, among women with BMI ≤ 25.0 the second item was green tea whereas, in the overweight women green tea was number 4. Among overweight women number two in the list was boiled coffee, whereas in the women with BMI ≤ 25.0 boiled coffee was number 4. Clementines were not on the list in women with BMI ≤ 25.0, but they were last in the list among overweight women. In both strata the food items mentioned above explained 98% of the variation in intake of antioxidants.

## Discussion

In this article we report the food items that contributed most to the variation in antioxidant intake among Norwegian women. Among these food items different types of coffee and various types of tea were found to be the most important. A few fruits, vegetables and berries/nuts also contributed modestly to the variation. Among fruits the most important item was oranges and among vegetables it was broccoli. However, once coffee and tea were removed from the list of top 20 food items contributing most to the variation in antioxidant intake, the remaining items only contributed around 22%. In other words, compared to the contribution of coffee and tea, fruits and vegetables only explained a modest amount of the between person variation in antioxidant intake.

Part of the reason why coffee is so important, may be that the coffee intake is high in Norway. Coffee consumption is quite high in Norway and the other Nordic countries as compared to the rest of the world [28]. According to the International Coffee Organization (ICO) 2012, the average per capita consumption was highest in Finland (11.7 kg/year), followed by Norway (9.4 kg), Denmark (8.9 kg) and Sweden (8.1 kg), while in other European countries it was somewhat lower: Switzerland (7.4 kg), Germany (6.8 kg), Austria (6.8 kg), Belgium (6.4 kg), Netherlands (6.3 kg), Italy (5.6 kg) and France (5.4 kg) [29].

While we found that coffee captured the largest variation in antioxidant intake, the study by Svilaas *et. al*. [20] with fewer food items reported coffee to be the main contributor to total antioxidant intake, with this item contributing 68% to the total antioxidant intake and tea contributed 22%. Thus, in addition to coffee being high in antioxidants and consumed by a large part of the population, it is also consumed in sufficient variation to contribute both to the total and to the variation in antioxidant intake.

According to the European Food Information Council (EUFIC), the mean vegetable intake (including pulses and nuts) in Europe is 220 g per day and mean fruit intake is 166 g per day, implying that the average consumption of fruit and vegetables is 386 g per day [30]. According to the European Food Safety Authority (2008) the highest mean intake of fruits and vegetables was in Poland, with 577 g/day followed by Italy (452 g/day), the lowest mean intake was in Iceland 196 g/day [30]. The mean intake in Norway as was reported in a nationwide survey held in 2010–11 (NORKOST 3 study) to be 387 g per day for women and 363 grams per day for men [31]. These results suggest that the intake of fruits and vegetables in the present study population is comparable to the rest of the European population. Our study sample had a mean intake of 538 g/day.

The intake of fruits and vegetables in the present study is considerable higher (538 g/day) than the mean intake in Norway reported in the nationwide survey (NORKOST 3 study) i.e. 387 g per day for women [31]. This may be because of the higher detail in our questionnaire than in the nationwide survey, or because our questionnaire had questions on seasonality that may have resulted in higher estimates than previously. Alternatively, our participants may have been particularly health conscious, as compared to the previous survey participants.

The dietary intake varies between age groups, however when we ran analyses stratified by age the list of food items capturing the variation in antioxidant intake did not vary substantially. Comparing the younger women with the ones who were older, the sequence of food items was more or less the same, but in slightly different order. Similarly, there was no difference in the list of food items explaining the variation in antioxidant intake according to BMI. This suggests that antioxidant intake did not vary much between the underweight and the obese women.

Governmental recommendations on intake of fruits and vegetables have many similarities, but also some differences across countries. There are also some differences in the definition of fruits and vegetables. Juice is sometimes excluded from the fruit and vegetable recommendations (e.g. Belgium, Spain), included with limitations (e.g. counts as maximum 1 portion (e.g. Denmark, the Netherlands, Sweden and Norway), and fully included in other countries (e.g. Iceland). In Norway, potatoes and starchy tubers (such as sweet potatoes, yams rich in carbohydrates) were previously included in the vegetable definitions, while most other countries (e.g. Austria, Belgium, Denmark, Iceland, Netherlands, Portugal, Spain and Sweden) have followed the World Health Organization (WHO) recommendations and not included potatoes and starchy tubers as vegetables [32]. However, in the most recent Norwegian dietary guidelines potatoes were not included in the vegetable definition [33].

Willet [19] described a procedure for identifying food items that ought to be followed when creating dietary questionnaires. A key element in this is to identify foods where intake varies between individuals. If everyone eats an orange a day this will not contribute to the between person variation in antioxidant intake. However, if those who consume a diet high in antioxidants do so by consuming rare foods (for example beetroots), then this may contribute to the between person variation.

In addition to assessing variation in the nutrients and foods that are important, epidemiologic studies also need a FFQ with sufficient questions to assess total energy. Individual variation in intake of food items with various nutrients produces differences in total energy intake between individuals. This results in a positive correlation between consumption of most nutrients and the total energy intake [19]. Total energy is associated with multiple diseases, and therefore often represents a confounder in the analysis of the association between nutrients and disease.

The results from this study suggest that a relatively short list of food items would capture almost 98% of the variation in antioxidant intake. This suggests that addressing the role of antioxidants in epidemiological studies is feasible and specifically that it is not necessary to include a long list of uncommonly eaten herbs, spices, and fruits. A similar exercise may be useful for researchers particularly interested in capturing the variation in another specific nutrient.

One of the strengths of this study is that we used a fairly large sample of women. Our questionnaire used was detailed, validated and comprehensive, hence capturing most of the food items rich in antioxidants. To our knowledge, apart from Willet who describes the between person variation in food intake (according to Willet “*for a food item to be informative it must have three characteristic; first it must be eaten by a considerable number of individuals. Second, the food must have a substantial amount of the nutrient of interest. Third, to be discriminating, the use of food must vary from person to person, to illustrate this, a question about carrots would not help to rank subjects according to carotene intake if everyone ate one carrot a day”)*[34], no previous published study has specifically addressed capturing the variation in antioxidant intake.

A limitation of our study was the relatively low response rate on the first question included in the NBCSP questionnaire, i.e. when we asked whether women would be interested in participating in a dietary survey. This may have led to our participants being healthier than the rest of the population. However, this is a limitation we share with most other studies, such as the national survey NORKOST 3, which had a participation rate of 37% [31]. Another limitation was the use of self-reported dietary intake which, even though we used a validated questionnaire, is prone to measurement error [35]. However, FFQ’s are considered to be an economical and practical method to monitor dietary intake variation in large populations [36,37]. Further, it is highly unlikely that the use of FFQ caused a bias in selection of dietary items that explain the variation in antioxidant intake. In addition we used a database based on only one method, the FRAP assay, to measure the antioxidant intake. However, this is the only assay that directly measures antioxidants or reductants in a sample. Compared to the other assays, another advantage is that FRAP does not detect glutathione or protein thiols, as these molecules are for a large part degraded in the intestine and poorly absorbed [38]. As glutathione is poorly absorbed by humans, and as almost no other antioxidant thiols are present in dietary plants except in garlic, the FRAP method may be suitable for assessment of total antioxidants in dietary plants [26]. Finally, the study was limited by including only women. It is possible that the findings may have been different in men, in particular with respect to intake of fruits, vegetables and beverages.

## Conclusions

In summary, in this study we identified a list of food items which capture the amount of antioxidant intake among Norwegian women age 50 to 69 years. The major contributors to dietary total antioxidant capacity were coffee, tea, red wine, blueberries, walnuts, oranges, cinnamon and broccoli. It is important that these items are assessed in as much detail as possible in studies that wish to capture the variation in antioxidant intake. Our study provides an example of how one can identify major sources of variation in nutrients that can ultimately then be used to design questionnaires that capture the variation in the nutrient of interest.

## Competing interests

The authors declare that they have no conflict of interests.

## Authors’ contributions

SAQ carried out the literature review, data analyses, interpreted the results, and drafted the manuscript. ACL helped with data cleaning, drafting and proof reading the manuscript. MBV helped with the analysis, drafting and proof reading of the manuscript. MHC, RB and LFA helped in developing and designing the FFQ, drafting and proof reading of the manuscript. GU designed the study and contributed as supervisor and provided all scientific and technical supports. All authors read and approved the final manuscript.

## Pre-publication history

The pre-publication history for this paper can be accessed here:

http://www.biomedcentral.com/1471-2458/14/45/prepub
